# Assessment of β-Thalassemia Trait Occurrence in an Outpatient Sample from Southern Chile: A Retrospective Study

**DOI:** 10.3390/diagnostics15212759

**Published:** 2025-10-31

**Authors:** Mario Balcázar-Villarroel, Angélica Mancilla-Uribe, Sandra Navia-León, Luis Carrasco-Fajardin, Johana Bohle-Matamala, Cristian Calbucura-Ovalle, Francisco Torrens, Eduardo Carmine-Peña, Cristian Sandoval, Francisca Peña

**Affiliations:** 1 Laboratorio Clínico TecnoMedic, Puerto Montt 5502901, Chile; mariobalcazar91@gmail.com (M.B.-V.); informaciones.tecnomedic@gmail.com (A.M.-U.); sandra.navia@gmail.com (S.N.-L.); gerencia.losalamos@gmail.com (L.C.-F.); jovibohle@yahoo.com (J.B.-M.); cristianfelipe03@gmail.com (C.C.-O.); 2 Institut Universitari de Ciència Molecular, Universitat de València, 46071 València, Spain; torrens@uv.es; 3 Carrera de Medicina, Facultad de Medicina, Universidad de La Frontera, Temuco 4811230, Chile; e.carmine01@ufromail.cl; 4 Escuela de Tecnología Médica, Facultad de Salud, Universidad Santo Tomás, Los Carreras 753, Osorno 5310431, Chile; 5 Departamento de Medicina Interna, Facultad de Medicina, Universidad de La Frontera, Temuco 4811230, Chile

**Keywords:** β-thalassemia trait, frequency, microcytosis

## Abstract

**Background/Objectives:** Thalassemia comprises a group of heterogeneous hereditary hemoglobinopathies characterized by impaired hemoglobin synthesis due to mutations in the α, β, and/or δ globin genes. The resulting ineffective erythropoiesis produces anemia of variable severity depending on the affected globin chain. Although β-thalassemia is most prevalent in the Mediterranean region, the Middle East, and Southeast Asia, migration has contributed to its global spread, including in non-endemic areas. In Chile, published data on β-thalassemia trait (BTT) and β-thalassemia major (BTM) remain scarce. This study aimed to estimate the frequency of BTT in referred outpatients to a clinical laboratory in southern Chile. **Methods:** A retrospective observational study was conducted between January 2021 and November 2024 at a clinical laboratory in Puerto Montt. Complete Blood Cell counts (CBCs) from unique patients were reviewed, and those confirmed with confirmed thalassemia (HbA2 > 3.5%) were selected. **Results:** During the study period, 24,634 CBCs were reviewed. Sixty patients were confirmed as carriers of BTT, corresponding to a frequency of 0.24% (CI 95%: 0.18–0.31%) in the referred outpatients to laboratory (60/24,634). This occurrence is higher than the only previously published Chilean estimate but lower than frequencies reported in several South American countries. **Conclusions:** This investigation demonstrates a relatively low but non-negligible frequency of BTT in outpatients from southern Chile. The findings emphasize the importance of considering BTT in the differential diagnosis of microcytic anemia, a condition often underestimated in routine practice. Broader multicenter studies across Chile are warranted to validate these results and to provide a clearer picture of the epidemiology of β-thalassemia in the country.

## 1. Introduction

Inherited hemoglobin (Hb) problems represent the most prevalent genetic blood disorders worldwide, contributing to roughly 3.4% of mortality in children under five years old. This collection of disorders results from mutations in human globin genes, categorized into two types: those that generate structurally aberrant globin (Hb variants) and those that exhibit compromised globin synthesis (thalassemia) [1].

Thalassemia represents one of the most widespread hereditary blood disorders worldwide [2]. It comprises a heterogeneous group of conditions resulting from mutations in the α-, β-, and/or δ-globin genes, which impair hemoglobin production and lead to ineffective erythropoiesis [3]. The severity of anemia depends on the specific globin chain involved. Clinically, thalassemias are divided into α-thalassemia, β-thalassemia, δβ-thalassemia, and rare forms such as γδβ-thalassemia, with α- and β-thalassemia being the most common [4].

Thalassemias are common in tropical and subtropical regions where malaria has been and continues to be endemic. The elevated prevalence may result from carriers of hemoglobinopathies possessing a survival advantage in regions endemic to malaria [5]. Individuals with thalassemia variations are predominantly found in Southeast Asia, the Mediterranean region, the Indian subcontinent, the Middle East, and Africa. Furthermore, it is significant because, due to recent extensive population migrations, thalassemia is no longer confined to typical high-incidence areas and has become a somewhat prevalent clinical issue in North America, Northern Europe, and Australia. The clinical management of thalassemia, including its diagnosis and treatment, has posed challenges to the local health system [6,7].

α-thalassemia is caused by diminished or absent synthesis of α-globin chains and is marked by microcytic hypochromic anemia. Individuals with mild α-thalassemia may be discovered inadvertently through microcytosis, whereas those with moderate-to-severe variants present a wide spectrum of clinical symptoms, from asymptomatic anemia to hydrops fetalis. The initial group is indicated to possess the α-thalassemia trait, whereas the two most clinically relevant diseases in the subsequent group are Hemoglobin H (HbH) disease and Hb Bart’s hydrops fetalis syndrome (BHFS) [8].

The variations in phenotypic severity are typically associated with the degree of imbalance between α- and non-α-globin chain production and the proportion of free α-chains. In α-thalassemia, the concentration of β-like chains exceeds that of α chains; conversely, in β-thalassemia, the concentration of β-like chains is inferior to that of α chains. The extent of imbalance correlates with the severity of the condition [9].

β-thalassemia is further classified into three categories: major (BTM), intermedia, and trait (BTT). Patients with BTM inherit two defective alleles and develop severe anemia, requiring lifelong transfusion support. Individuals with β-thalassemia intermedia also carry two abnormal alleles but experience a milder clinical course compared with BTM. In contrast, BTT results from a single defective allele and is generally asymptomatic [10]. The principal predictor of β-thalassemia severity is the type of β allele (β^0^, β^+^, β^++^), influenced by the co-inheritance of interacting α-thalassemia and the intrinsic capacity to enhance γ chain synthesis [11]. Numerous strategies have been developed to mitigate the pathogenic imbalance of the α/β ratio to diminish surplus free α-globin by either decreasing the α-globin chain, reinstating β-globin expression, or reactivating γ-globin expression, resulting in diminished disease severity, reduced treatment requirements and intervals, and fewer complications, thereby enhancing patient quality of life and alleviating economic burdens [12].

It is estimated that approximately 1.5% of the global population, equivalent to 80–90 million individuals, carry β-thalassemia, with around 60,000 affected children born each year, the majority in developing countries [13]. The overall incidence of symptomatic cases has been reported at about 1 per 100,000 worldwide, and 1 per 10,000 within the European Union. Despite these figures, robust epidemiological data on carrier prevalence remain limited, particularly in regions known or expected to bear a high disease burden [14,15]. Historically, β-thalassemia has shown its highest prevalence in the Mediterranean basin, the Middle East, and Southeast Asia. Nevertheless, due to population migration, its presence is increasingly recognized in non-endemic regions [16]. The highest carrier frequencies of β-thalassemia have been documented in Cyprus (14.0%), Sardinia (10.3%), and several regions of Southeast Asia [14]. This distribution is thought to be associated with the selective advantage conferred against *Plasmodium falciparum* malaria [17]. Global migration and interethnic marriages have subsequently facilitated the spread of thalassemia to nearly all parts of the world, including Northern Europe, where it was previously absent. Over 350 unique mutations have been recognized as causative factors for β-thalassemia. Approximately 20 variants account for over 80% of cases globally, a phenomenon linked to geographical clustering, where each population generally contains a limited number of common mutations alongside a range of less frequent ones [18].

In Latin America, thalassemia has been described as a cause of microcytic anemia, with its frequency varying according to the genetic background of each country’s population, including wide differences in carriage within the same country [19,20]. In this regard, current migration patterns, especially from Venezuela to other countries [21], could have an impact on the prevalence of thalassemia in the region, given the natural reproduction of offspring among the migrant and native populations. In addition to its heterogeneous prevalence among different South American countries, thalassemia has been described as an underdiagnosed cause of microcytic anemia, often confused with iron deficiency anemia (IDA) [22], probably due to its relative infrequency in most countries, coupled with the relatively high cost and low availability of hemoglobin electrophoresis as a test used for screening or confirmation.

With respect to our country, information available in Chile is still limited. Although the diagnosis of BTT is made by medical specialists, few reports have documented isolated instances of BTT [23,24,25,26] and BTM [27], along with the application of discriminatory indices for differential diagnosis [28,29]. In the field of epidemiology, a single study has indirectly assessed the frequency of BTT carriers [30], while another has provided age-standardized prevalence rates related to the global burden of disease [2].

This retrospective study sought to ascertain the frequency of BTT among referred outpatients to our clinical laboratory in Puerto Montt and surrounding areas of southern Chile over a nearly four-year timeframe, addressing the lack of national data.

## 2. Materials and Methods

A retrospective observational search was performed in the Laboratory Information System (LIS, ProActive©, Christchurch, New Zealand) of the referred outpatients to our clinical laboratory located in Puerto Montt (Tecno-Medic), between January 2021 and November 2024, to identify those patients with confirmed BTT, that is, a hemoglobin A_2_ (HbA_2_) fraction > 3.5% [31]. To observe the hematological phenotype, Complete Blood Count (CBC) results were collected; to assess iron metabolism, blood tests were reviewed; and to evaluate morphologic characteristics, blood smears were stained. Initially, 27,503 CBCs were determined, but after filtering to eliminate duplicate patients, the total number of CBCs from outpatients referred to our clinical laboratory was 24,634.

The following examinations were performed to determine whether patients fulfilled the inclusion criteria:

CBC: Venous blood samples were obtained in ethylenediaminetetraacetic acid dipotassium (EDTA-K_2_) anticoagulant tubes. CBCs, encompassing red blood cell, white blood cell, hemoglobin, hematocrit, and platelet measurements, were evaluated utilizing the Mindray BC-5380 automated hematology analyzer (Mindray Medical International Limited, Shenzhen, China), in accordance with the manufacturer’s guidelines. Daily quality control procedures were conducted with standardized commercial controls supplied by the manufacturer.

Iron metabolism: Serum samples were collected after overnight fasting and analyzed on the Mindray BS-480 fully automated chemistry analyzer (Mindray Medical International Limited, Shenzhen, China). The following parameters were reviewed: serum iron (Fe), total iron-binding capacity (TIBC), and unsaturated iron-binding capacity (UIBC). Transferrin saturation (TSAT) was calculated as the ratio of serum iron to TIBC, expressed as a percentage. Assays were performed using manufacturer-provided reagents and protocols, with calibration and internal quality controls applied daily to ensure analytical accuracy and precision. The following reference values (RV) were considered for diagnosing concomitant IDA in patients with BTT: in adult men Hb < 13 g/dL and Fe < 65 ug/dL plus TSAT < 16%; in adult women Hb < 12 g/dL and Fe < 50 ug/dL plus TSAT < 16%, and in children (<15 years) Hb < RV according to age and Fe < 30 ug/dL plus TSAT < 16% [32,33,34].

Ferritin: Serum samples were collected after overnight fasting and analyzed using the Maglumi-800 fully automated chemiluminescence immunoassay analyzer (Snibe Diagnostic, Shenzhen, China). The assay is based on a sandwich chemiluminescence immunoassay (CLIA) principle, employing specific anti-ferritin antibodies labeled with acridinium ester. Serum samples were processed according to the manufacturer’s instructions, and calibration was performed with reference standards provided by the manufacturer. Internal quality controls at both low and high concentration levels were run daily to ensure analytical reliability. The following RV were considered for diagnosing concomitant IDA in patients with BTT: in adult men: Hb < 13 g/dL and ferritin < 28 ng/mL; in adult women Hb < 12 g/dL and ferritin < 12 ng/mL, and in children (<15 years): Hb < RV according to age and ferritin < 12 ng/mL [32,33,34].

Hemoglobin electrophoresis: Venous blood samples were obtained in ethylenediaminetetraacetic acid dipotassium (EDTA-K_2_) anticoagulant tubes. Hemoglobin electrophoresis was performed at an external clinical laboratory in Santiago, Chile. Hemoglobin fractions were separated based on their electrophoretic mobility, and quantification of HbA_2_ was carried out using capillary electrophoresis performed on the analyzer (Capillarys 2 Analyzer, Sebia, Lisses, France). HbA_2_ fraction > 3.5% was considered diagnostic of BTT [31]. Assays were performed using manufacturer-provided reagents and calibrators, with internal quality controls applied daily to ensure analytical accuracy and precision.

Morphological evaluation: Peripheral blood smears were prepared from EDTA-anticoagulated samples, stained with May-Grünwald–Giemsa (Merck, Darmstadt, Germany), and examined under oil immersion at 100× magnification. Smears were evaluated by an experienced Medical Technologist for red blood cell morphology, including anisopoikilocytosis, hypochromia, microcytosis and RBC inclusions, following international guideline recommendations [35]. In this sense, basophilic stippling was defined as the occurrence of fine, medium, or coarse blue granules due to abnormally aggregated ribosomes, uniformly distributed throughout the RBC; therefore, we considered that the observation of this inclusion in at least one erythrocyte constituted our criterion for positivity. Quality control was ensured by systematic review of reference slides with known morphological abnormalities.

## 3. Results

Between January 2021 and November 2024, a total of 27,503 CBCs were performed. After eliminating patients with more than one CBC performed, the final number was 24,634 CBCs. Of these, 60 cases were confirmed as BTT (HbA_2_ > 3.5%), corresponding to a frequency of 0.24% (60/24,634, CI; Confidence Intervals 95%: 0.18–0.31%). Demographic and hematological data were extracted from the LIS (Table 1).

**Figure 1:**
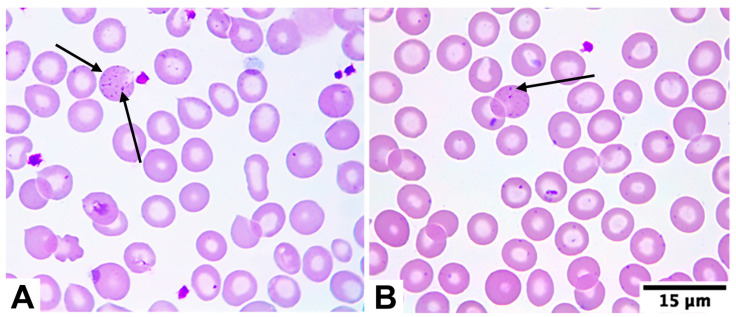
Patients with confirmed BTT exhibit basophilic stippling (black arrows in (**A**,**B**)). This inclusion is usually found in low proportion of RBCs in blood smear, so a thorough search should be performed in patients suspected of having BTT.

The mean age of affected individuals was 44.0 years (SD, standard deviation: 23.2 years), with a predominance of females (68.3%, 41/60) compared to males (31.7%, 19/60). Based on the World Health Organization (WHO) age- and sex-adjusted cut-off values for hemoglobin [34], 58.3% (35/60) of BTT patients presented anemia, whereas 41.7% (25/60) had hemoglobin levels within the normal range. In none of the 60 patients was IDA present. Furthermore, among the 60 patients confirmed with BTT, basophilic stippling was observed in 53 of them (88.3%; 53/60) (Figure 1).

## 4. Discussion

β-thalassemia is one of the most common hereditary disorders worldwide, with a heterogeneous clinical presentation. It is characterized by impaired hemoglobin synthesis caused by mutations in the β-globin gene and/or the β-LCR (β locus control region), leading to ineffective erythropoiesis and anemia of variable severity [36]. The prevalence of the disease differs across regions, with the highest rates observed in countries surrounding the Mediterranean, the Middle East, and Southeast Asia [6].

In the present study, the frequency of BTT was estimated at 0.24% (CI 95%: 0.18–0.31%), which is higher than the 0.06% (11 cases among 18,206 patients) previously reported in the only other Chilean study, conducted in the capital city [30]. These discrepancies could reflect differences in inclusion or exclusion criteria as well as potential regional variation in prevalence between northern and southern Chile, separated by nearly 1000 km. However, this latter hypothesis cannot be confirmed due to the lack of additional epidemiological studies in other parts of the country. We believe that a theory with a certain degree of support for the higher frequency found in our cohort could be the greater genetic diversity of the southern Chilean population, influenced by significant waves of German immigration to the Llanquihue Lake basin during the mid-19th to early 20th centuries [37]. BTT has been identified as the most frequent thalassemia type in individuals of German descent, with reported prevalence rates as high as 13.7% in large-scale studies of native populations [38]. Future investigations in southern Chile would also be of interest in regions shaped by different migratory flows, such as the far south, where immigration during the same historical period was predominantly Croatian [39]. Notably, Croatia reports a BTT prevalence of approximately 0.8% [40], which is lower than that documented in Germany but nearly fourfold higher than the rate observed in our current Chilean cohort. In any case, these theories should be considered as hypotheses-generating since, unfortunately, we do not have studies on the prevalence of BTT in other parts of Chile or population genetic studies that would allow us to establish this relationship with certainty.

### 4.1. South American Comparison

Given the absence of comprehensive national data on BTT in Chile, comparison with reports from other South American countries provides a useful perspective. In Brazil, the overall prevalence of thalassemia (BTT and BTM combined) has been estimated at 1.0%, with regional variation observed across the country [20]. In Argentina, Chiappe described a BTT carrier prevalence ranging between 1% and 2% [41]. Similarly, Arends et al. reported a prevalence of 1.48% in Venezuela, either as isolated BTT or in association with Sickle cell hemoglobin (HbS) [42], while data from Colombia indicated a nationwide prevalence of 18.4%, again with regional differences [19]. In Peru, a frequency of 2.5% was documented, either as isolated BTT or in combination with other hemoglobinopathies [43]. Table 2 presents frequencies in other South American countries. It is noteworthy that, for the remaining countries, no data on BTT and/or BTM frequencies were available.

Taken together, these findings indicate that Chile exhibits lower frequencies of BTT compared with neighboring countries, except when compared to Ecuador, and practically the same as for Uruguay. Of particular importance are the prevalence reported for Venezuela, Colombia, and Peru, as migrants from these countries currently account for 62.5% of the foreign-born population residing in Chile [46]. This demographic trend suggests that the prevalence of BTT in Chile may rise in the future due to intermarriage and generational transmission. Such circumstances also carry the risk of homozygous thalassemia, given a 25% probability of inheritance when both parents are carriers.

These considerations highlight the potential need for national strategies addressing thalassemias, including screening programs, prenatal diagnostic approaches, and genetic counseling. Such policies, already implemented in countries with higher prevalence, have contributed to mitigating the global burden of morbidity and mortality associated with these disorders [2].

### 4.2. Hematological Phenotype

With regard to the hematological phenotype of the patients diagnosed with BTT in our study, the findings showed slight differences compared with those described by Laensgri et al. in Thailand [47], but were largely consistent with the results reported by Grau et al. in Spain [48], except for RDW-CV values. This discrepancy is not unexpected, since RDW-CV is known to have a low degree of inter-laboratory standardization and is subject to considerable variability depending on the analyzer used [49]. The overall similarity with the Spanish cohort may reflect the historical impact of Spanish colonization across South America from the 16th century onward, coupled with the minimal genetic influence from Southeast Asian populations in Chile.

A notable observation was the relatively high proportion of individuals without anemia (41.7%), based on WHO sex- and age-specific hemoglobin thresholds [34]. Although many published reports suggest that non-anemic patients are included in BTT cohorts, these data are rarely disaggregated to estimate their relative frequency. However, our findings align with those of Kulkarni et al., who described 44.5% of pregnant women with BTT in India as non-anemic [50], and Mazza et al., who reported 54.0% of BTT carriers without anemia in Italy [51]. Similarly, Wickramaratne and Wijewickrama found that 4.5% of Sri Lankan carriers had hemoglobin ≥ 14 g/dL, leading them to caution against the exclusive use of hemoglobin levels for BTT screening, as this approach could fail to detect a significant proportion of carriers [52].

The variability in hematological indices among BTT patients has been extensively studied, with consensus that erythrocyte parameters are strongly influenced by the type of underlying mutation—whether β^0^ mutations (complete absence of β-globin chain synthesis) or β^+^ mutations (partial reduction in β-globin production) [53,54].

### 4.3. Morphological Characteristics

With respect to morphological features, basophilic stippling was observed in 88.3% (53/60) of BTT patients (Figure 1). This rate was higher than those reported by Vayá et al. (80.0%) [55], Körber et al. (72.7%) [56], Aixalá (72.3%) [57], Lazarte et al. (67.0%) [58], and Harrington et al. (16.7%) [59], but lower than the frequencies described by Calero et al. (96.0%) [60] and Schriever (100.0%) [61].

Although basophilic stippling can occur in various conditions [62], our findings support the view that, when detected within the context of microcytosis and hypochromia—with or without anemia, and generally in small proportion of RBCs—it is strongly suggestive of BTT. This interpretation is consistent with observations from other authors [61,63]. Consequently, targeted smear evaluation by medical technologists remains essential after reviewing CBC results in combination with the patient’s clinical history.

### 4.4. Clinical Implications

The findings of this study emphasize the importance of considering BTT in the differential diagnosis of microcytic anemias. Misclassification of BTT as IDA remains a common clinical challenge [22]. Accurate identification through combined evaluation of hematological indices, iron metabolism parameters, and confirmatory tests such as hemoglobin electrophoresis enables more precise treatment decisions. First, it avoids iron therapy for patients who do not require it and determines effective treatment with folic acid for patients with BTT who require it (1–5 mg daily). However, this treatment is not essential for patients who consume normal amounts of raw vegetables and/or fruits, except in cases of pregnancy, infections, and major surgeries [64]. Second, it enables genetic counseling for patients with BTT, explaining to the carrier patient that if they have children with someone who has hemoglobinopathy, there is a 25% chance of having offspring with severe clinical conditions and a 50% probability of transmitting BTT to their offspring if the couple has a normal phenotype [64]. Third, the use of some discriminatory index with great demonstrated accuracy (for example, Green & King index) [28,29] as screening could increase diagnosis, considering the limited ability of non-specialist physicians to suspect the presence of BTT based on CBC data. In this sense, in United States, Hansen et al. [65] demonstrated that 68% of patients suspected of having BTT did not consider this possibility. On the other hand, in Israel, Shalev et al. [66] reported that only 27% of patients with suggestive CBCs had a definitive diagnosis of BTT. Similarly, in India, Kakkar et al. [67] reported that 84% of patients suspected of having BTT did not consider this possibility, and only 7.1% achieved a definitive diagnosis. In Latin America, Ruiz [68] reported that BTT is underdiagnosed and frequently confused with IDA in Mexico. Given this background, Table 3 shows the most common and frequently available laboratory tests to make a correct differential diagnosis.

### 4.5. Public Health Implications

**Figure 2:**
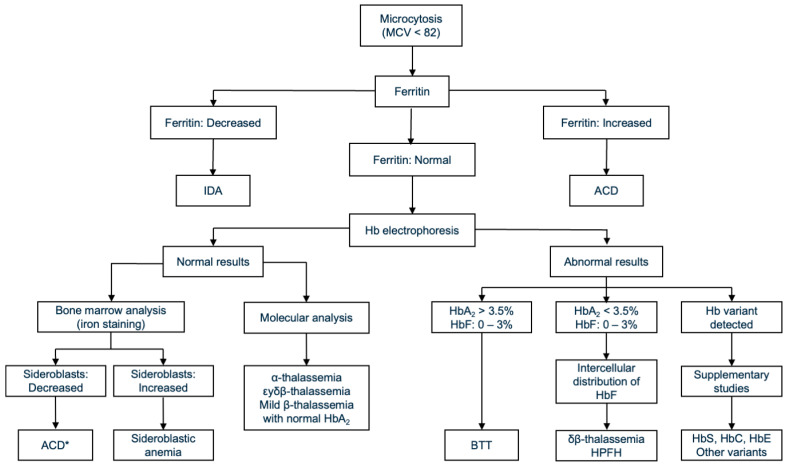
Flowchart for differential diagnosis in microcytic anemias or microcytosis without anemia (modified from references [69,70]). * Since bone marrow analysis is an invasive and generally poorly tolerated procedure, the diagnosis of ACD may be based on other laboratory tests, along with a thorough clinical evaluation if there is an underlying pathology.

From a public health perspective, the recognition of BTT in the Chilean population underscores the need for enhanced awareness and systematic screening approaches. The absence of standardized diagnostic protocols increases the risk of underdiagnosis, leading to inefficient resource use and missed opportunities for preventive interventions. Integrating targeted thalassemia screening into routine laboratory practice could improve diagnostic accuracy, optimize healthcare resources, and contribute to the development of evidence-based national guidelines. Furthermore, strengthening professional training on hemoglobinopathies at the primary care and hematology levels would facilitate earlier detection and better patient outcomes. In this sense, we propose a diagnostic scheme summarized in Figure 2, which should be adapted according to the technology available for each health center.

### 4.6. Limitations

Several limitations of this study should be acknowledged. The most evident is the relatively small number of confirmed BTT cases in our database from unique patients (*n* = 60), which contrasts with the larger sample sizes reported in other international studies [51,60]. This can be attributed to the modest scale of our clinical laboratory, the population size of Puerto Montt and its surrounding areas, and the low frequency of BTT previously described in Chile compared with countries within the so-called “thalassemia belt” [2,30].

Regarding the possibility of having missed the opportunity to confirm patients with CBCs suggestive of BTT but who did not have hemoglobin electrophoresis results available, given the retrospective and observational nature of our study and considering that it is not a population-based survey, but rather a laboratory-based descriptive study, we are aware that this possibility exists. However, given the training that has been carried out in our setting with clinical staff regarding microcytic anemia based on our previous studies [28,29], added to the fact that in CBCs suggestive of BTT our laboratory reports the Green and King index, given its good performance demonstrated in a small cohort of our own patients [28] and in other studies [71,72,73,74], we believe that this omission had a minimal influence, and the frequency that could have been obtained by us would have varied slightly. This assumption is further supported by the fact that the frequency observed in this study was four times higher than that reported in the only prior Chilean investigation (0.24% vs. 0.06%) [30].

Finally, another limitation was the lack of evaluation of possible confounding effects from α-thalassemias and/or δβ-thalassemia, which have been considered in other studies [75]. Although these conditions have not been described in Chile to date, likely due to the complexity of molecular diagnosis, particularly for α-thalassemias, they have been reported in neighboring countries such as Argentina [76]. Thus, the future appearance of clinically relevant cases of native origin in Chile cannot be excluded.

## 5. Conclusions

This study identified a relatively low frequency of BTT in referred outpatients to a clinical laboratory in southern Chile, representing one of the first investigations of its kind in the country. These findings contribute new evidence for the region and highlight the relevance of including BTT in both clinical and public health agendas. Future multicenter studies with larger and more diverse cohorts across different regions of Chile are warranted to confirm prevalence estimates, refine diagnostic strategies, and inform national health policies aimed at improving the detection and management of thalassemia traits.

## Figures and Tables

**Table 1:** Summary of demographic and hematological parameters of patients confirmed with BTT.

Parameter	Mean	SD	Minimum	Maximum
Age (years)	44.0	23.2	1	95
RBC (×10^6^/mm^3^)	5.94	0.58	5.00	7.41
HB (g/dL)	12.2	1.20	10.0	15.3
HCT (%)	38.6	3.83	31.9	49.3
MCV (fL)	64.9	3.13	57.4	70.9
MCH (pg)	20.5	1.06	17.8	22.6
MCHC (%)	31.5	0.66	30.3	33.2
RDW-CV (%)	14.5	0.79	13.0	18.0
PLT (×10^3^/mm^3^)	291.3	75.94	130.0	493.0
MPV (fL)	9.2	0.82	7.6	12.3
HbA2 (%)	4.8	0.49	3.6	6.4
Green & King index *	50.64	6.25	36.2	68.5

HB: hemoglobin; HCT: hematocrit; MCH: mean corpuscular hemoglobin; MCHC: mean corpuscular hemoglobin concentration; MCV: mean corpuscular volume; MPV: mean platelet volume; PLT: platelet count; RBC: red blood cell count; RDW-CV: red cell distribution width—coefficient variation; SD: standard deviation; * Green & King index: (MCV × MCV × RDW-CV)/(HB × 100), value < 65.0 is suggestive of BTT and value > 65.0 is suggestive of IDA.

**Table 2:** Summary of prevalence of β-thalassemia trait in countries of South America.

Country	Prevalence (%)	BTT Patients *	Sample Size	Characteristic of the Sample Population	Confirmatory Method	Ref.
Colombia	18.4%	409	2224	Outpatient cohort (adults and children)	Hb ELP	[19]
Brazil	1.00%	87	8715	Outpatient cohort (only adults)	HPLC	[20]
Chile	0.06%	11	18,206	Outpatient cohort (only adults)	Hb ELP	[30]
Argentina	1–2%	NR	NR	NR	NR	[41]
Venezuela	1.48%	1189	80,400	Outpatient cohort (age not mentioned)	Hb ELP and HPLC	[42]
Peru	2.50%	130	5206	Outpatient cohort (age not mentioned)	Hb ELP	[43]
Ecuador	0.0%	0	115	Outpatient cohort (only adults)	Hb ELP	[44]
Uruguay	0.25%	1	397	Outpatient cohort (only children)	Hb ELP and molecular confirmation	[45]

* β-thalassemia trait with or without hemoglobinopathies (for more details, please refer to each reference); NR: not reported; Hb ELP: hemoglobin electrophoresis; HPLC: High-Performance Liquid Chromatography.

**Table 3:** Common laboratory tests for the differential diagnosis of microcytic anemias.

Test	IDA	ACD	BTT	α Thalassemia	BTT + IDA
Serum iron	Decreased	Normal/decreased	Normal	Normal	Decreased
TIBC	Increased	Normal/decreased	Normal	Normal	Increased
TSAT	Decreased	Normal/decreased	Normal	Normal	Decreased
Ferritin	Decreased	Increased	Normal	Normal	Decreased
HbA2	Normal/decreased	Normal	Increased	Normal/decreased	Normal/decreased
HbF	Normal	Normal	Normal/increased	Normal	Normal/decreased/increased

IDA: Iron deficiency anemia; ACD: Anemia of chronic disease; BTT: β-thalassemia trait; TIBC: Total iron binding capacity; TSAT: % Transferrin saturation. Modified from reference [64].

## Data Availability

The original contributions presented in this study are included in the article. Further inquiries can be directed to the corresponding authors.
